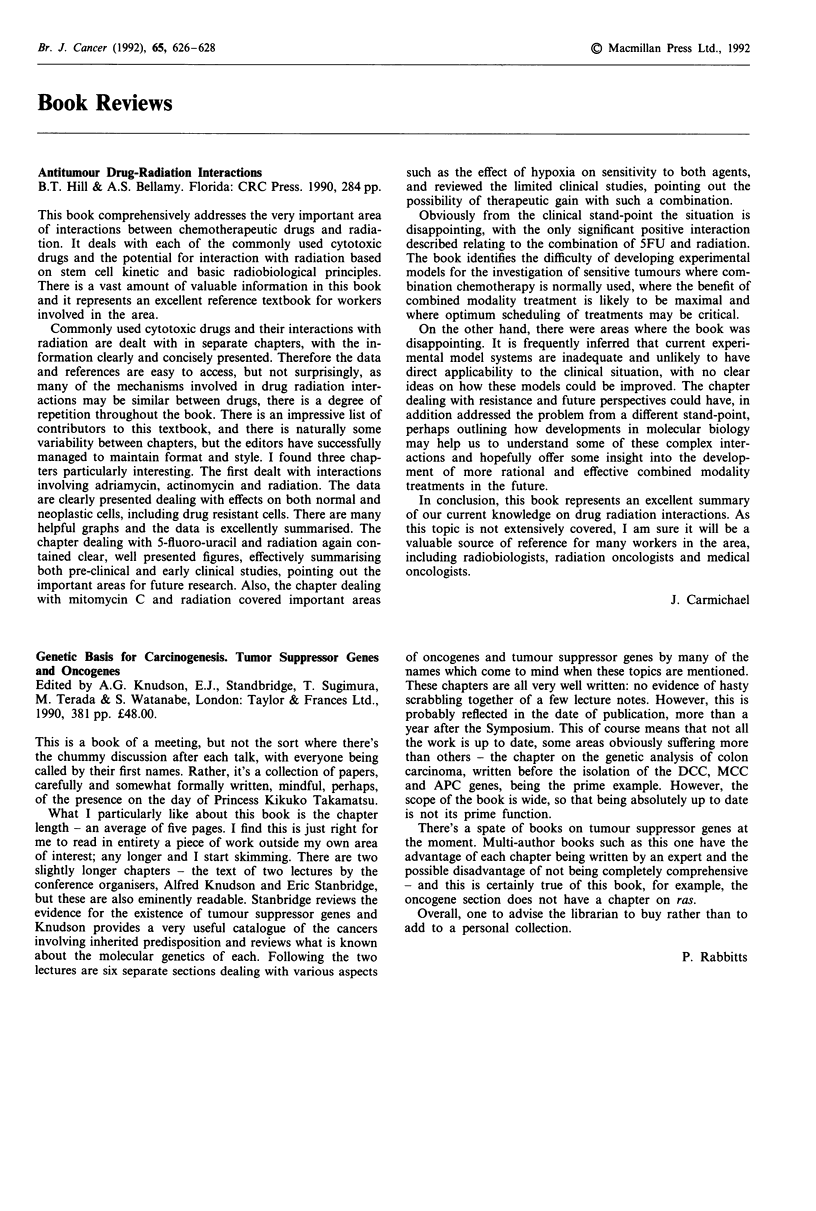# Antitumour Drug-Radiation Interactions

**Published:** 1992-04

**Authors:** J. Carmichael


 © Macmillan Press Ltd., 1992

### Book Reviews

**Antitumour Drug-Radiation Interactions**
B.T. Hill & A.S. Bellamy. Florida: CRC Press. 1990, 284 pp.

This book comprehensively addresses the very important area of interactions between chemotherapeutic drugs and radiation. It deals with each of the commonly used cytotoxic drugs and the potential for interaction with radiation based on stem cell kinetic and basic radiobiological principles. There is a vast amount of valuable information in this book and it represents an excellent reference textbook for workers involved in the area.

Commonly used cytotoxic drugs and their interactions with radiation are dealt with in separate chapters, with the information clearly and concisely presented. Therefore the data and references are easy to access, but not surprisingly, as many of the mechanisms involved in drug radiation interactions may be similar between drugs, there is a degree of repetition throughout the book. There is an impressive list of contributors to this textbook, and there is naturally some variability between chapters, but the editors have successfully managed to maintain format and style. I found three chapters particularly interesting. The first dealt with interactions involving adriamycin, actinomycin and radiation. The data are clearly presented dealing with effects on both normal and neoplastic cells, including drug resistant cells. There are many helpful graphs and the data is excellently summarised. The chapter dealing with 5-fluoro-uracil and radiation again contained clear, well presented figures, effectively summarising both pre-clinical and early clinical studies, pointing out the important areas for future research. Also, the chapter dealing with mitomycin C and radiation covered important areas such as the effect of hypoxia on sensitivity to both agents, and reviewed the limited clinical studies, pointing out the possibility of therapeutic gain with such a combination.

Obviously from the clinical stand-point the situation is disappointing, with the only significant positive interaction described relating to the combination of 5FU and radiation. The book identifies the difficulty of developing experimental models for the investigation of sensitive tumours where combination chemotherapy is normally used, where the benefit of combined modality treatment is likely to be maximal and where optimum scheduling of treatments may be critical.

On the other hand, there were areas where the book was disappointing. It is frequently inferred that current experimental model systems are inadequate and unlikely to have direct applicability to the clinical situation, with no clear ideas on how these models could be improved. The chapter dealing with resistance and future perspectives could have, in addition addressed the problem from a different stand-point, perhaps outlining how developments in molecular biology may help us to understand some of these complex interactions and hopefully offer some insight into the development of more rational and effective combined modality treatments in the future.

In conclusion, this book represents an excellent summary of our current knowledge on drug radiation interactions. As this topic is not extensively covered, I am sure it will be a valuable source of reference for many workers in the area, including radiobiologists, radiation oncologists and medical oncologists.

J. Carmichael